# Identification and characterization of transcript polymorphisms in soybean lines varying in oil composition and content

**DOI:** 10.1186/1471-2164-15-299

**Published:** 2014-04-23

**Authors:** Wolfgang Goettel, Eric Xia, Robert Upchurch, Ming-Li Wang, Pengyin Chen, Yong-Qiang Charles An

**Affiliations:** 1 USDA-ARS, Midwest Area, Plant Genetics Research Unit at Donald Danforth Plant Science Center, 975 N Warson Rd, St. Louis, MO 63132, USA; 2 508 East Stoughton Street, Champaign, IL 61820, USA; 3 USDA-ARS, Soybean and Nitrogen Fixation Research, 2417 Gardner Hall, Raleigh, NC 27695, USA; 4 USDA-ARS, Plant Genetic Resources Conservation Unit, 1109 Experiment St., Griffin, GA 30223, USA; 5 Department of Crop, Soil and Environmental Sciences, University of Arkansas, Fayetteville, AR 72701, USA

## Abstract

**Background:**

Variation in seed oil composition and content among soybean varieties is largely attributed to differences in transcript sequences and/or transcript accumulation of oil production related genes in seeds. Discovery and analysis of sequence and expression variations in these genes will accelerate soybean oil quality improvement.

**Results:**

In an effort to identify these variations, we sequenced the transcriptomes of soybean seeds from nine lines varying in oil composition and/or total oil content. Our results showed that 69,338 distinct transcripts from 32,885 annotated genes were expressed in seeds. A total of 8,037 transcript expression polymorphisms and 50,485 transcript sequence polymorphisms (48,792 SNPs and 1,693 small Indels) were identified among the lines. Effects of the transcript polymorphisms on their encoded protein sequences and functions were predicted. The studies also provided independent evidence that the lack of *FAD2-1A* gene activity and a non-synonymous SNP in the coding sequence of *FAB2C* caused elevated oleic acid and stearic acid levels in soybean lines M23 and FAM94-41, respectively.

**Conclusions:**

As a proof-of-concept, we developed an integrated RNA-seq and bioinformatics approach to identify and functionally annotate transcript polymorphisms, and demonstrated its high effectiveness for discovery of genetic and transcript variations that result in altered oil quality traits. The collection of transcript polymorphisms coupled with their predicted functional effects will be a valuable asset for further discovery of genes, gene variants, and functional markers to improve soybean oil quality.

## Background

Soybean [*Glycine max* (L.) Merrill] is the largest oil crop in the US. Soybean seed oil composition and content are important agronomic traits, determining nutritional value as well as utility for biodiesel production and other industrial applications. A number of key enzymes important for producing storage lipids in oilseed species have been identified [1]. Studies of developing seeds and/or embryos have suggested that the biosynthetic pathways for fatty acids and lipids are largely regulated at the transcriptional level [2-4].

The most common genetic variations in eukaryotes are single nucleotide polymorphisms (SNPs) [5]. Cultivated soybean and its wild ancestor *Glycine soja* have an estimated average SNP frequency of one SNP per 1,000 bp and one SNP per 425 bp of contiguous genome sequence, respectively [6]. Short DNA insertions and deletions also contribute to intra-species genomic variation. Structural variants resulting from chromosome breaks and repairs, include large-scale chromosomal rearrangements such as inversions, translocations, duplications, large insertions and deletions. Presence/absence variations (PAV) and copy number variations (CNV) have also been shown to play important roles in phenotypic variation. PAV and CNV are defined as gains or losses of DNA segments usually larger than 1 kb that often contain one or more genes [7]. They are prevalent in plant genomes and have been described in soybean as well [7-9]. Epigenomic variations, which cause phenotypic diversity in the absence of sequence alterations, are also reflected in changes of gene expression [10]. The epigenomes of soybean recombinant inbred lines have recently been analyzed [10,11]. Genetic variations with functional significances are transcribed into transcript sequence and expression variations, which eventually lead to phenotypic diversity. Identification of transcript sequence and expression variations in oil quality related genes would thus facilitate the discovery of functional variations and accelerate soybean oil quality improvement.

The advent of next generation sequence technologies (NGS) has provided an efficient means to simultaneously determine transcript sequences and expression levels on a genome scale. RNA-seq offers unique advantages compared to whole genome sequencing. Although the soybean genome is 1.1 Gb in size, transcribed sequences account for no more than 100 Mb of the entire genome. Accordingly, RNA-seq reduces the effective genome size and also the associated costs of sequencing to approximately 10% of whole genome sequencing. Although non-transcribed regulatory genome sequences cannot be captured by RNA-seq, their regulated products, transcript accumulation levels can be measured by RNA-seq. Thus, RNA-seq can effectively determine two functional attributes of a gene, transcript sequence and accumulation level. Sequence and expression polymorphisms associated with gene functions could potentially be identified as underlying causes of phenotypic variation.

In soybean research, RNA-seq has been applied mainly in determining accumulation of coding and non-coding RNAs in a single cultivar [12-17], while whole genome sequencing has been used for SNP discovery, phylogenetic and population genetic studies [18-22]. Although RNA-seq has recently been utilized in plant species for SNP discovery [23-28], a comprehensive and in-depth characterization and functional annotation of transcript sequence and expression polymorphisms is not yet available for any plant species. As a proof-of-concept, we sequenced seed transcriptomes at a mid-maturation stage of nine soybean lines varying in oil composition and content. We developed and applied a variety of bioinformatic analysis algorithms to identify the sequence and expression variations (transcript polymorphisms) among the genotypes, and further predicted their effects on gene function. The transcript polymorphisms of the genes in acyl-lipid related pathways were further characterized to identify transcript polymorphisms potentially leading to changes in oil composition and content among the genotypes. We demonstrated that RNA-seq is a fast and cost-effective approach to detect and characterize sequence and expression variations in soybean lines. The collection of transcript polymorphisms provides a valuable resource for discovery of gene and gene variants potentially controlling important oil quality traits and the other seed traits.

## Results

### Soybean genotypes varying in seed oil composition and content

**Figure 1:**
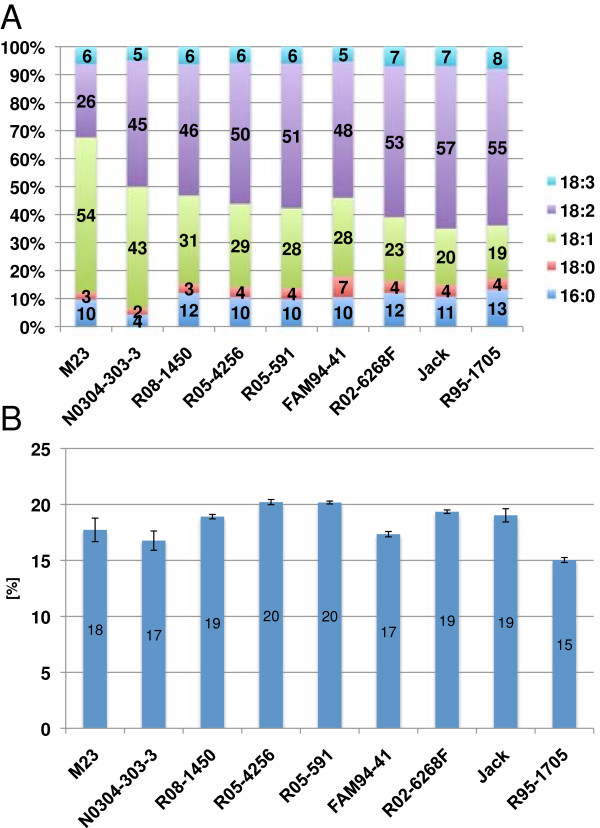
**Oil-composition and content of seeds for each soybean line. (A)** The fatty acid composition of soybean seeds is shown for all nine soybean lines used in this study. The major fatty acids are palmitic acid (16:0), stearic acid (18:0), oleic acid (18:1), linoleic acid (18:2), and linolenic acid (18:3). The number before the colon indicates the number of carbon atoms, and that after the colon, the number of double bonds in the fatty acid chain. Lines are sorted by their oleic to linoleic acid ratio. Commodity soybean oil typically contains 13% palmitic acid (16:0), 4% stearic acid (18:0), 20% oleic acid (18:1), 55% linoleic acid (18:2), and 8% linolenic acid (18:3) [29]. **(B)** The oil content of soybean seeds is presented for all nine soybean genotypes used in this study.

Nine soybean genotypes varying in oil composition (Figure 1A) and content (Figure 1B) were selected for RNA sequencing. Under our growth condition, R95-1705 and N0304-303-3 represented low oil content genotypes that contained 15.0% and 16.8% of oil, respectively. Both R05-591 and R05-4256 accumulated 20% oil in their seeds and represented high oil content genotypes. Commodity soybean oil is typically composed of 13% palmitic acid (16:0), 4% stearic acid (18:0), 20% oleic acid (18:1), 55% linoleic acid (18:2), and 8% linolenic acid (18:3) [29]. The oil composition in some examined lines dramatically differed from that of conventional commodity soybean lines. For example, N0304-303-3 had the lowest palmitic acid content with 4.3%. FAM94-41 contained 7.3% of stearic acid, which was significantly higher than that in commodity soybean lines. M23 and N0304-303-3 were mid-oleic acid genotypes with 54.5% and 42.7%, respectively, and had the lowest amount of linoleic acid with 25.9% and 44.7%, respectively. N0304-303-3 produced the lowest quantity of linolenic acid at 4.6%.

### RNA-Sequencing and transcript profiles in soybean seeds

Transcriptomes of soybean seeds at the mid-maturation stage were sequenced. On average, 35 million 100 nt long pair-end sequencing reads were generated for each genotype (Table 1). Sixty-seven percent of the sequence reads aligned uniquely to the reference soybean genome of Williams 82 [30]. The total length of transcribed sequences was 72 million nucleotides on average, and accounted for 6.5% of the soybean genome (Table 1). An average of 73% of those transcribed sequences aligned to gene models. The mean coverage for transcribed genome sequences and gene models was 21 and 27, respectively. A total of 33,779 genes, which included 32,885 previously annotated gene models, were transcribed in seeds (Table 1). Thus, 60.7% of the 54,175 gene models were expressed in soybean seeds.

**Table 1:** Summary of RNA-seq datasets

**Soybean line**	**Jack**	**FAM94-41**	**M23**	**N0304-303-3**	**R02-6268 F**	**R05-4256**	**R05-591**	**R08-1450**	**R95-1705**	**Total**	**Average**
Total no. of sequenced reads (in million)	31.2	37.3	38.7	31.1	45.3	28.1	35.1	35.1	29.3	311.2	34.6
No. of reads aligned uniquely (in million)	20.6	25.9	25.1	19.9	31.7	17.1	23.3	25.2	19.8	208.6	23.2
Percent reads aligned uniquely	66.1	69.5	64.9	63.9	70.0	61.0	66.6	71.7	67.7	-	66.8
Total length of transcripts covered (in million nt)	63.5	76.4	70.2	65.1	80.8	66.3	77.4	75.9	72.5	130.5	72.0
Total length of annotated transcripts covered (in million nt)	49.7	54.7	53.0	49.7	57.2	49.9	55.4	52.0	52.0	69.7	52.6
Average depth of coverage per transcript nt	20.0	20.8	19.7	19.8	24.7	17.9	19.3	24.8	19.1	-	20.7
Average depth of coverage per annotated transcript nt	24.6	27.7	25.1	24.9	33.4	22.7	25.7	34.4	25.4	-	27.1
No. of genes identified (in thousand)	33.3	33.3	33.4	33.3	33.4	33.3	33.4	33.0	33.3	33.8	33.3
No. of annotated genes identified (in thousand)	32.6	32.6	32.6	32.6	32.7	32.5	32.6	32.2	32.5	32.9	32.5

**Figure 2:**
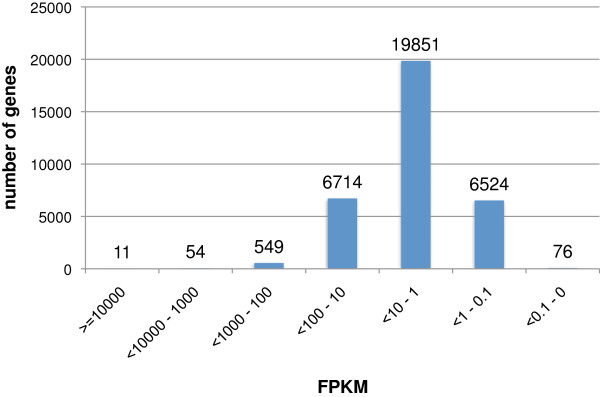
**Expression distribution.** Expression among genes can vary by several orders of magnitude. The numbers of genes per expression interval as indicated on the X-axis are displayed on the Y-axis.

Figure 2 shows the distribution of transcribed genes with respect to their transcript accumulation levels. While gene expression varied over several orders of magnitude, the average and median expression levels were 194.2 and 31.8 FPKM (Fragments per kilo- base of transcript per million mapped reads) respectively. Most of the genes were expressed at levels between 0.1 to 100 FPKM. 0.2% of genes were highly expressed at more than 1,000 FPKM. 21.5% of genes had a medium expression level ranging from 1,000 to 10 FPKM while 78.3% were expressed at low levels of less than 10 FPKM (Figure 2). The top 3 and 79 most abundant transcripts accounted for 11% and 50% of all transcripts accumulating in seeds, respectively (Additional file 1: Figure S1).

Table 2 summarizes functions and accumulation levels of the 79 most abundant gene transcripts, which accounted for 50% of the total RNA population in soybean seeds. The most abundant transcript in seeds accumulated at 33,706 FPKM, accounting for 4.6% of all transcripts, encoded a 2S albumin precursor (Protease inhibitor/seed storage/LTP family) (Additional file 2: Table S1). The highly abundant seed transcripts encode a variety of well-characterized proteins such as seed storage proteins, protease inhibitors, proteases, acyl lipid enzymes, oil body proteins and late embryogenesis abundant (LEA) proteins, and as well proteins of unknown functions. Ten of the 79 most abundant genes encoded seeds storage proteins, which included four of six functional glycinin genes and four of eight β-conglycinin genes [31,32]. Collectively, they amounted to almost one fifth of the total seed transcripts. Eleven highly abundant transcripts encoded enzymes and structural proteins involved in acyl lipid metabolism. They included the three lipoxygenases genes, *Lox1*, *Lox2* and *Lox3*[33] and the fatty acid desaturase gene, *FAD2-1B*, which encodes one of two enzymes converting oleic acid to linoleic acid. In addition, they contain six oleosin genes and one caleosin gene that function in oil body biogenesis and degradation [34-37].

**Table 2:** Most abundant transcripts in seeds

**Categories**	**No. of genes**	**Expression [%]**	**Examples**
Seed storage proteins	10	19.2	glycinins, conglycinins, 2S albumins
Protease inhibitors	3	4.1	Bowman-Birk serine protease inhibitor, Kunitz trypsin inhibitor
Proteases	4	3.9	
Acyl lipid enzymes	4	3.0	lipoxygenases, fatty acid desaturase
Oil body proteins	7	2.8	oleosins, caleosins
Seed maturation proteins/late embryogenesis abundant proteins/dehydrins	17	4.6	
Metallothioneins	3	1.4	
Aquaporin-like proteins	3	0.6	
Ribosomal protein	2	0.3	
Lectins	2	0.8	
ADP-ribosylation factor A1F	2	0.4	
Miscellaneous	11	4.8	defensin, leginsulin 1, ADR6 auxin down-regulated, elongation factor 1-alpha, allergen Gly m Bd 28 K, alcohol dehydrogenase 1, S-adenosylmethionine synthetase, transcription activator-related…
Uncharacterized proteins/proteins of unknown function	11	4.3	
	79	50.1	

### Identification and characterization of transcript expression polymorphisms

Out of 33,779 genes identified in at least one of the nine lines, 31,909 genes (94.5%) were expressed in all nine genotypes (Table 3). 1,870 genes were transcribed in one to eight lines, of which the majority (975 genes) was expressed in eight lines. Interestingly, 47 genes were only expressed in any one of the lines (Table 3). The median transcript accumulation value of the genes expressed specifically in one line was 131.3 FPKM, and was higher than that of genes expressed in multiple lines. These genes encoded proteins with a variety of biological functions and proteins with unknown functions. It will be interesting to investigate the functions of those line-specific transcripts and their contribution to the phenotypic diversity.

**Table 3:** Presence-absence variation in nine lines

**No. of lines**	**Common genes**	**Mean expression**	**Median expression**
1	47	131.28	8.05
2	70	16.34	3.93
3	64	190.57	2.51
4	104	11.32	2.17
5	107	5.58	1.48
6	155	24.31	1.26
7	348	4.52	1.10
8	975	28.30	0.85
9	31909	19.93	3.65

A total of 8,037 genes showed significant variations in their expression levels among the nine lines based on a Z-score cutoff at +/-2 (Additional file 2: Table S2). R08-1450 and Jack, which contained the highest number of genes exhibiting significant expression variation, had 3,357 and 1,478 genes, respectively (Additional file 1: Figure S2). Other lines had 268 to 657 genes exhibiting expression variation. More genes showed increased expression over reduced expression (55 vs 45%). Two hundred and seventy genes that had mean expression values equal to or higher than 10 FPKM showed at least a 10-fold difference in their transcript accumulation (Additional file 2: Table S3). For example, transcripts of Glyma06g04740, a Gibberellin-regulated family protein gene, accumulated 318 times less in R08-1450 than its average in all lines.

### Transcript expression polymorphisms of putative acyl lipid genes

A total of 1,090 genes potentially involved in acyl lipid metabolic and signaling pathways were identified in the soybean genome (Table 4) based on their sequence similarity with the annotated acyl lipid genes in Arabidopsis [30,38]. The genes were categorized into nine functional classes [30]. Seventy-four percent of the putative acyl lipid genes were expressed in seeds. They included approximately 90% of genes involved in the synthesis of fatty acids in plastids, the synthesis of membrane lipids in endomembrane systems, the metabolism of acyl lipids in mitochondria, and the synthesis and storage of oil (Table 4). In contrast, only 45% of genes participating in the fatty acid elongation and wax and cutin metabolism were expressed in seeds.

**Table 4:** Expression variation of putative acyl lipid genes

**Function category of acyl lipid genes**	**Acyl lipid genes**	**Expressed in seeds**	**Diff. expressed**	**Percent expr. in seeds**	**Percent diff. expressed**
Synthesis of fatty acids in plastids	78	70	11	89.74	15.71
Synthesis of membrane lipids in plastids	54	31	13	57.41	41.94
Synthesis of membrane lipids in endomembrane system	101	89	19	88.12	21.35
Metabolism of acyl lipids in mitochondria	33	30	5	90.91	16.67
Synthesis and storage of oil	36	31	4	86.11	12.90
Degradation of storage lipids and straight fatty acids	142	104	29	73.24	27.88
Lipid signaling	295	191	52	64.75	27.23
Fatty acid elongation and wax and cutin metabolism	84	38	11	45.24	28.95
Miscellaneous	267	185	44	69.29	23.78
Total	1090	769	188	73.87	24.05

One hundred eighty-eight genes, accounting for 24% of soybean acyl lipid genes, showed variations in their transcript accumulation levels in seeds (Table 4). A Fisher’s exact test revealed that the functional category “synthesis of membrane lipids in plastids” was significantly overrepresented among genes with expression polymorphisms at a p-value of 0.042. There was no overrepresentation of genes with expression polymorphisms in any other acyl lipid functional category at a p-value cutoff of 0.05. Examples for genes with expression polymorphisms included the *FAD3A* gene (Glyma14g37350) (Additional file 2: Table S2). The *FAD3A* gene encodes a linoleate desaturase in the “Synthesis of membrane lipids in plastids” category. While the FPKM and Z-score values for *FAD3A* were 12. 1 and -2.31 in N0304-303-3, respectively, the average FPKM value for the remaining eight lines was 43.57 FPKM. The 3.6 fold decrease in expression was correlated with a decrease in linolenic acid levels (Figure 1A). *FAD3A* was the most abundant among the three gene transcripts encoding linoleate desaturases, whose products convert linoleic acid into linolenic acid. The other two homologs, *FAD3B* (Glyma02g39230) and *FAD3C* (Glyma18g06950), had an average accumulation value of 27.88 and 11.33 FPKM, respectively. It is likely that the lower expression of *FAD3A* reduces accumulation of linolenic acid levels in N0304-303-3, but the residual *FAD3A* expression in N0304-303-3 combined with the *FAD3B* and *FAD3C* expression still result in a substantial amount of linolenic acid in seeds.

### Identification and characterization of transcript sequence polymorphisms

We identified a total of 48,792 SNPs in the nine lines in reference to the Williams 82 genome at a read depth cut-off of 5 reads for each SNP (Table 5). An average of 23,669 SNPs were present in each line with a mean coverage of 16 reads per SNP in annotated genes. Jack had the lowest number of SNPs with 15,752; and R08-1450 had the highest number of SNPs with 28,962. R08-1450 also had the highest number of genes with expression polymorphisms (see above). A comparison of our SNP data with those in the Single Nucleotide Polymorphism database (dbSNP) (http://www.ncbi.nlm.nih.gov/projects/SNP/) showed that about 82% of the SNPs in annotated genes had previously been identified, which offered an independent confirmation.

**Table 5:** SNP annotation

**Soybean line**	**Jack**	**FAM94-41**	**M23**	**N0304-303-3**	**R02-6268 F**	**R05-4256**	**R05-591**	**R08-1450**	**R95-1705**	**Total**	**Average**
Total SNP count in annotated genes	15,752	27,123	24,906	24,909	26,504	19,308	21,040	28,962	24,518	48,792	23,669
Average read depth per SNP	13	17	15	14	20	12	15	21	15	-	16
Total length of annotated transcripts covered (in nt)	49,680,448	54,713,084	53,001,316	49,673,932	57,245,339	49,901,790	55,367,363	51,984,636	51,682,304		
SNPs overlapping with dbSNP	12,748	22,182	20,494	20,380	21,741	15,598	17,082	23,828	20,048	39,998	19,345
SNPs in annotated exons	14,271	23,757	22,450	22,311	23,202	17,023	18,333	25,381	21,544	43,283	20,919
SNPs in annotated UTRs	4,156	7,226	6,659	6,657	6,939	5,082	5,533	7,644	6,479	12,748	6,264
SNPs in annotated CDS	10,115	16,531	15,791	15,654	16,263	11,941	12,800	17,737	15,065	30,535	14,655
Non-synonymous SNPs	4,759	7,814	7,441	7,338	7,769	5,704	6,113	8,383	7,271	14,372	6,955
Synonymous SNPs	5,365	8,731	8,366	8,329	8,512	6,250	6,704	9,372	7,808	16,196	7,715
SNPs eliminating start codons	6	11	9	8	8	7	7	12	6	17	9
SNPs causing premature termination codons	29	70	68	67	80	44	44	72	55	125	59
SNPs eliminating termination codons	24	28	29	31	30	21	23	29	28	49	27
SNPs in splice sites	220	369	342	323	354	245	254	357	313	606	309
SNPs in annotated introns	2,120	4,483	3,469	3,609	4,377	3,036	3,575	4,752	3,957	7,488	3,709

**Figure 3:**
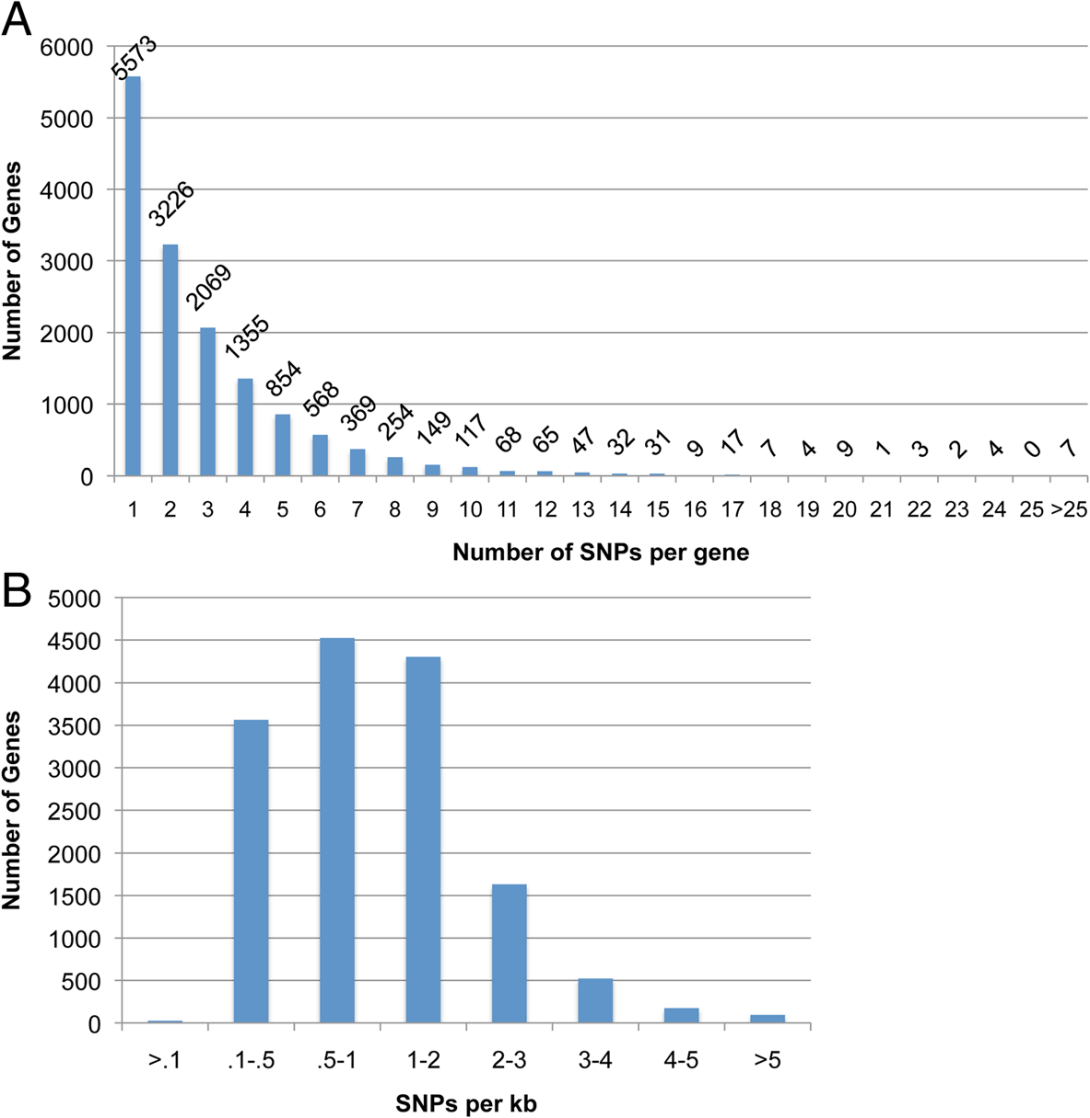
**Distribution of SNPs per gene and per kb of transcribed gene sequence. (A)** The distribution of the number of SNPs per gene is shown. Only 63 genes each contain more than 15 SNPs. **(B)** Intervals of varying sizes for SNP density given as number of SNPs per kb of transcribed gene sequence are indicated on the X-axis. The corresponding gene count is plotted on the Y-axis.

On average, we identified 1 SNP per 1,429 nt of transcript sequence. Forty-four percent of the 33,779 genes expressed in seeds contained SNPs (Figure 3A). The genes with and without transcript accumulation polymorphism had similar percentage of SNPs, suggesting no or little correlation between transcript SNPs and transcript accumulation polymorphism. The largest group of those genes (38%) had a single SNP. The largest bin of genes (30.5%) showed a SNP density between 0.5 and 1 SNP per kb length (Figure 3B). Only 93 genes contained more than 5 SNPs per kb.

To identify SNPs that potentially lead to changes in protein functions, SNPs in coding regions were categorized into non-synonymous (14,372) and synonymous (16,196) SNPs (Table 5). We detected 125 non-synonymous SNPs that change an amino acid codon into a stop codon, 49 non-synonymous SNPs that change a stop codon into an amino acid codon, and 17 SNPs that eliminate a start codon. In addition, we found 606 SNPs in splice sites that may affect transcript splicing and possibly protein sequence and function. Transcripts containing premature termination codons are often degraded by the nonsense-mediated decay (NDM) pathway [39]. We compared accumulation levels of transcripts in genotypes containing genes with and without premature stop codon. The average accumulation ratio of transcripts with and without the premature stop codon was 1.04, suggesting that there was no or little correlation between transcript accumulation and presence of a premature stop codon (Additional file 2: Table S4).

### Distribution of expressed genes and transcript SNPs within and among chromosomes

**Figure 4:**
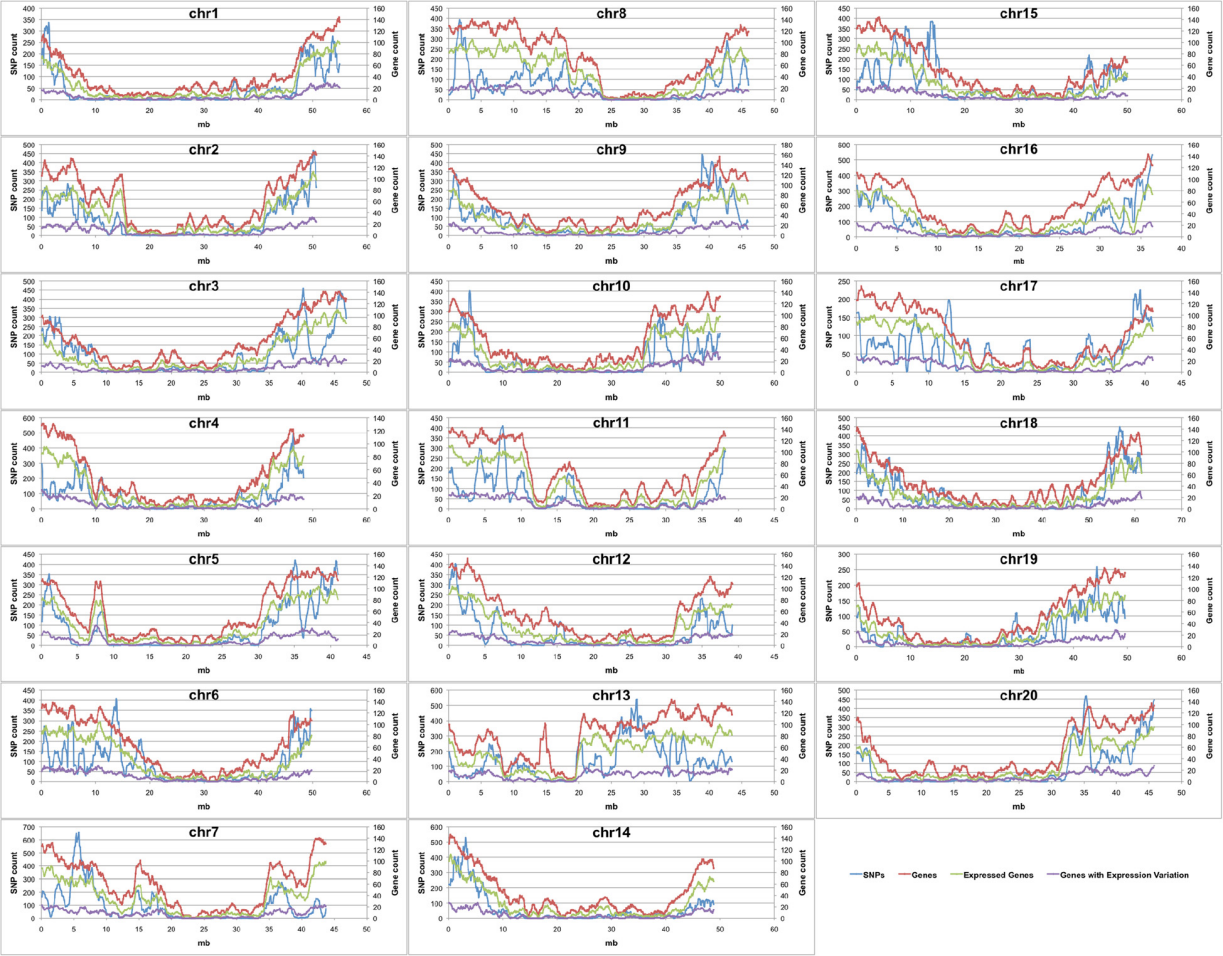
**Gene and SNP distribution.** To obtain the chromosomal distribution of genes, expressed genes, genes showing expression variation, and SNPs, we scanned each of the 20 soybean chromosomes by 1 mb sliding windows with 0.1 mb steps. SNP counts per 1 mb window are shown on the primary Y-axis and gene counts are displayed on the secondary Y-axis. The X-axis represents the chromosome length in mb.

The overall distribution patterns of genes were similar among most of the soybean chromosomes. Gene density was higher at both chromosome ends, but lower in the intervening regions (Figure 4). However, we also observed variations in the distribution patterns for a number of chromosomes. For example, the gene-rich regions expanded from the chromosomal ends towards the middle more on chromosomes 9 and 14. Discrete gene-rich regions can also be found in chromosomes 7, 12 and 18.

Overall, the distribution of expressed genes resembled that of genes along each chromosome. While on average, 61% of annotated genes were expressed in seeds, several chromosomal regions were found with highly different percentages of transcribed genes (Figure 4). For example, 85% of annotated genes located between positions 42.2-43.3mb on chromosome 4 were expressed in seeds. In contrast, only 21.3% of genes were transcribed in the region between positions 14–16.3 mb on chromosome 13. The highest densities per chromosome varied between 95 and 112 expressed genes per 1 mb. It has been reported that gene expression in pericentromeric regions is lower than in chromosomal arms [12,40,41]. Interestingly, our preliminary data indicated that there was no correlation between expression levels and chromosomal positions of expressed genes. Genes showed similar expression levels along chromosome ends and intervening regions. There was no obvious difference in the distribution of genes with and without expression polymorphisms (Figure 4).

The distribution patterns of the 48,792 identified SNPs along chromosomes are shown in Figure 4. As expected, SNPs and expressed genes had similar distribution patterns. The SNPs densities were often lower at center regions and higher at both chromosomal ends. However, on some chromosome regions, the number of SNPs per expressed gene varied dramatically from the average. Unlike gene and expressed gene densities, maximum SNP densities per chromosome varied almost 3 fold from 656 SNPs per 1 mb window on chromosome 7 to 225 SNPs per 1 mb window on chromosome 17.

### Characterization of transcript sequence polymorphism related to acyl lipid signaling and metabolic pathways

A total of 1,186 SNPs were identified in putative acyl lipid genes (Table 6). The distribution of those SNPs among all lines was similar to that of all SNPs; Jack and R08-1450 had the lowest and the highest number of SNPs, respectively. Among the nine lines, 708 SNPs were located in CDS, of which 428 were synonymous and 280 non-synonymous SNPs (Table 6). Non-synonymous SNPs cause a modification of protein sequences that can be benign or deleterious and possibly experience selection. The non-synonymous to synonymous SNP (N/S) ratio in acyl lipid genes was 0.65 while the N/S ratio in all examined genes was 0.89, suggesting that the protein sequence changes were more likely subjected to a negative selection in acyl lipid genes than in the other genes. In addition, we observed that one non-synonymous SNP in an E1-E2 ATPase gene (Glyma06g47300) caused a premature termination codon. Another non-synonymous SNP in Jack abolished a start codon in a protease inhibitor/seed storage/lipid transfer protein gene (Glyma03g04370.2). Five SNPs coincided with splice sites and might change protein sequences and functions.

**Table 6:** SNPs in putative acyl lipid genes

**Soybean line**	**Jack**	**FAM94-41**	**M23**	**N0304-303-3**	**R02-6268 F**	**R05-4256**	**R05-591**	**R08-1450**	**R95-1705**	**Total**	**Average**
Total SNP count in annotated genes	414	668	615	618	633	427	467	722	578	1186	571.33
SNPs in annotated exons	379	585	550	548	553	392	413	625	501	1012	505.11
SNPs in annotated UTRs	112	188	162	164	177	127	142	192	168	304	159.11
SNPs in annotated CDS	267	397	388	384	376	265	271	433	333	708	346.00
Non-synonymous SNPs	111	157	156	149	155	100	99	172	146	280	138.33
Synonymous SNPs	156	240	232	235	221	165	172	261	187	428	207.67
SNPs eliminating start codons	1	0	0	0	0	0	0	0	0	1	0.20
SNPs causing premature termination codons	1	1	1	1	0	1	1	1	1	1	0.89
SNPs eliminating termination codons	0	0	0	0	0	0	0	0	0	0	0.00
SNPs in splice sites	1	3	2	2	3	1	0	2	2	5	1.78
SNPs in annotated introns	56	114	88	96	98	58	81	126	103	174	91.11
SNPs overlapping with dbSNP	355	552	524	514	545	363	393	614	489	998	483.22

**Figure 5:**
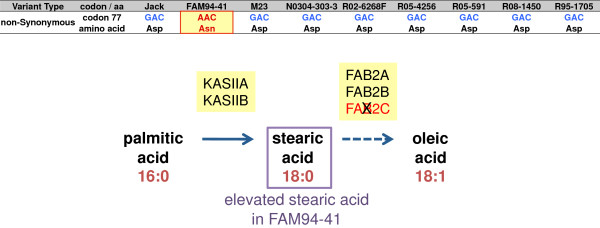
**Deleterious effects of a non-synonymous SNP in *FAB2C*.***FAB2C* (Glyma14g27990) encodes one out of three stearoyl-acyl-carrier-protein desaturases that convert stearic acid into oleic acid. The *FAB2C* allele in FAM94-41 contains the variant nucleotide G instead of the reference nucleotide A in the first nucleotide position of codon 77. This non-synonymous SNP results in an amino acid change from the acidic polar aspartic acid (Asp, encoded by GAC) to the polar asparagine (Asn, encoded by AAC), which renders the protein non-functional. FAM94-41 exhibits an elevated stearic acid phenotype as less stearic acid is converted into oleic acid.

We tested the putative effect of the non-synonymous SNPs on protein function by using the prediction tools PROVEAN [42], and PolyPhen-2 [43]. Based on a subset of SNPs in 102 randomly selected acyl lipid genes, 29% of non-synonymous SNPs were predicted to have deleterious effects on protein function (Additional file 2: Table S5). One of the non-synonymous SNPs was located in the *FAB2C* gene (Glyma14g27990), which encodes one out of three stearoyl-acyl-carrier-protein desaturases expressed in seeds. This SNP altered codon 77 from GAC to AAC, and consequently caused an amino acid change from aspartic acid (Asp, an acidic polar aa) to asparagine (Asn, a polar aa) (Figure 5). This SNP was only present in line FAM94-41. This *FAB2C* mutation in FAM94-41 is likely to result in the increased stearic acid level seen in Figure 1A [44,45]. As both homologous genes *FAB2B* (Glyma02g15600) and *FAB2A* (Glyma07g32850) are functional in FAM94-41, it is likely that stearic acid can still be converted to oleic acid although at a lower rate. Interestingly, we observed that accumulation of the *FAB2C* transcript was reduced in FAM94-41 (190.1 FPKM vs the average of 433.1 FPKM) while the transcript accumulation of *FAB2A* (42.2 FPKM vs the average of 35.4 FPKM) and *FAB2B* (205.3 FPKM vs the average of 175.4 FPKM) were increased in FAM94-41. The increased *FAB2A* and *FAB2B* expression may be able to partially compensate for the reduction of FAB2C activity in FAM94-41.

The transcript sequencing data also allow discovery of small, one and two bp long indels in addition to large genomic deletions (see below). We identified 1,693 of small indels in annotated genes, of which 1,365 indels were located in annotated exons (Table 7). In contrast to SNPs, 82.3% of the exonic indels were situated in UTRs while 17.7% of the small indels were located in coding sequences. The 241 indels in coding sequences were expected to change reading frames and amino acid sequences, and were likely to be deleterious to protein function. We also found 4 small indels in termination codons and 35 in splice sites. Thirty-four percent of indels identified in this study were reported in dbSNP.

**Table 7:** INDEL annotation

**Soybean line**	**Jack**	**FAM94-41**	**M23**	**N0304-303-3**	**R02-6268 F**	**R05-4256**	**R05-591**	**R08-1450**	**R95-1705**	**Total**	**Average**
Total INDEL count in annotated genes	660	1096	935	985	1115	803	891	1106	975	1693	951.78
INDELs in annotated exons	553	926	801	838	933	673	742	921	801	1365	798.67
INDELs in annotated UTRs	419	728	635	674	743	520	564	739	634	1124	628.44
INDELs in annotated CDS	134	198	166	164	190	153	178	182	167	241	170.22
INDELs in termination codons	2	3	3	3	2	1	3	3	3	4	2.56
INDELs in splice sites	16	33	26	30	29	20	29	25	25	35	25.89
INDELs in annotated introns	140	221	179	192	233	166	195	238	220	328	198.22
INDELs overlapping with dbSNP	243	365	335	341	380	298	326	409	345	578	338.00

A total of 45 small indels were found in 39 putative acyl lipid genes (Table 8). However, most of them were located in UTRs, and only 4 indels were present in CDS of three genes. For example, a Thioesterase/thiol ester dehydrase-isomerase gene (Glyma07g32710) showed a 1 bp homozygous insertion (T) in the first codon in lines M23, N0304-303-3, R02-6268 F, R05-4256 and R08-1450. A peroxygenase 2 gene (Glyma20g34300) contained a 1 bp heterozygous (T) and a 2 bp homozygous deletion (CT) in codons 118 and 141 in Jack, respectively.

**Table 8:** INDELs in putative acyl lipid genes

**Soybean line**	**Jack**	**FAM94-41**	**M23**	**N0304-303-3**	**R02-6268 F**	**R05-4256**	**R05-591**	**R08-1450**	**R95-1705**	**Total**	**Average**
Total INDEL count in annotated genes	16	22	18	21	20	13	10	24	20	45	18.22
INDELs in annotated exons	10	18	14	15	17	11	9	20	14	32	14.22
INDELs in annotated UTRs	7	16	12	13	15	9	8	18	13	28	12.33
INDELs in annotated CDS	3	2	2	2	2	2	1	2	1	4	1.89
INDELs in termination codons	0	0	0	0	0	0	0	0	0	0	0.00
INDELs in splice sites	0	0	0	0	0	0	0	0	0	0	0.00
INDELs in annotated introns	7	6	5	7	4	3	2	5	7	13	5.11
Homozygous INDELs	14	21	15	19	18	11	7	18	18	43	15.67
Heterozygous INDELs	2	1	3	2	2	2	3	6	2	16	2.56
INDELs overlapping with dbSNP	5	2	6	5	2	3	1	4	6	10	3.78

### Increased expression of a gene cluster in the *Rhg1* locus of Jack

**Figure 6:**
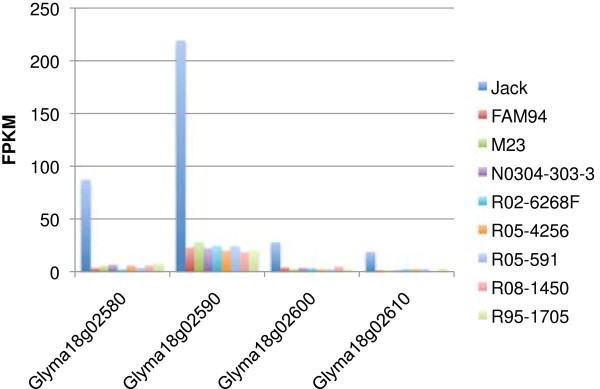
**Transcript analysis of a highly expressed multi-gene segment in Jack.** The adjacent genes Glyma18g02580, Glyma18g02590, Glyma18g02600 and Glyma18g02610 are about 15.5, 9.6, 8.0 and 9.0 times higher expressed in Jack than in all remaining lines, respectively. These genes compose the *Rhg1* locus that refers resistance to soybean cyst nematodes (SCN) when highly expressed.

We identified a segment consisting of four adjacent genes (Glyma18g02580, Glyma18g02590, Glyma18g02600 and Glyma18g02610), whose transcript accumulations were 15.5, 9.6, 8.0 and 9.0 times higher in Jack than their average expression level in the other eight lines, respectively (Figure 6). Interestingly, these genes were located in the previously characterized *Rhg1* locus (for resistance to *Heterodera glycines*). It has been reported that overexpression of these genes confers resistance to the soybean cyst nematode (SCN) *Heterodera glycines*[8]*.* Jack is resistant to SCN [46]. The co-ordinate elevated expression of the four genes is likely to lead to its SCN resistance. It has been shown that the copy numbers of a 31.2 kb fragment containing the four genes vary greatly in soybean lines, and their transcript accumulation is correlated with their copy number [8]. The SCN resistant Fayette cultivar has 10 copies while the susceptible Williams 82 cultivar has just one copy of this 31.2 kb fragment. We conducted shotgun sequencing of the Jack genome at a 10-fold average coverage (unpublished). Interestingly, the coverage for each of the four *rhg1* genes was between 101 and 113 reads, indicating a 10 to 11-fold copy number increase of these genes. This suggests that the amplification of the *rhg1* genes leads to their increased transcript accumulation and SCN resistance in Jack.

### Assessment of genetic diversity among the nine genotypes

**Figure 7:**
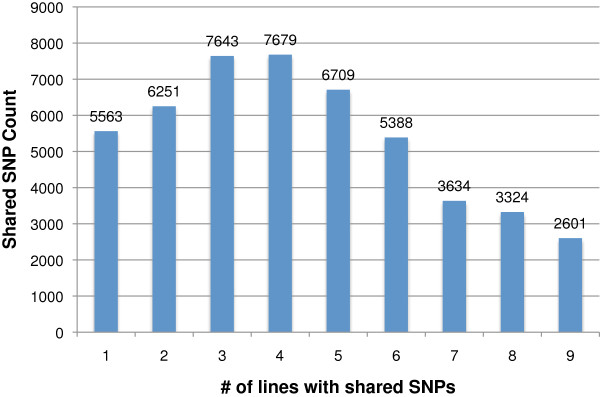
**Common SNPs among lines.** SNPs are unique or shared among lines. For each of the 48792 SNP, we determined the number of lines that carry the variant nucleotide compared to Williams 82. The total number of shared SNPs is shown as a function of the number of lines containing the SNP.

We determined the presence of variant nucleotides at congruent SNP positions in all nine soybean lines. A total of 5,563 SNPs, which accounted for 11.4% of all discovered SNPs, were line-specific (Figure 7). Jack had the highest number with 1,255 SNPs while R05-4256 had the lowest number with 23 SNPs to the minor allele frequency (MAF) SNPs. The remaining 43,229 SNPs were shared by two to nine lines. A total of 7,679 (15.7%) SNPs were present in four lines representing the largest set among shared SNPs. Two thousand six hundred one (5.3%) SNPs were common in all nine lines indicating that the reference genome Williams 82 carries the unique variant nucleotide compared to the nine lines.

**Figure 8:**
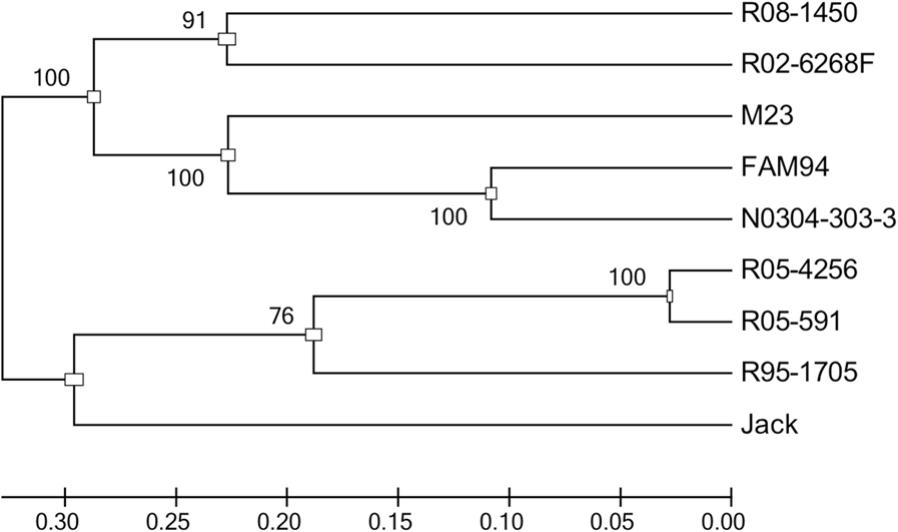
**Maximum Likelihood tree of nine soybean lines.** The Maximum Likelihood tree is based on 33,164 out of 48,792 SNP positions found in all nine lines compared to Williams82. SNP positions that contained heterozygous SNPs, missing nucleotides (lines with no read coverage) or identical nucleotides in all lines were excluded from the analysis. The Bootstrap method with 1,000 replications was used to test the phylogeny. The Bootstrap values are shown at the tree nodes. The scale bar indicates the number of nucleotide substitutions per site.

We assembled nine pseudo molecules consisting only of nucleotides at all SNP positions. Their alignments were used to infer the phylogenetic relationship among the nine lines. We generated a Maximum Likelihood tree based on 33,164 nucleotide SNPs (Figure 8). The mean genetic distance was 0.548 nucleotide substitutions per site. Jack and R08-1450, the lines with pronounced expression differences, were placed on divergent branches. R05-4256, R05-591 and R95-1705 formed a clade. FAM94-41, N0304-303-3 and M23 grouped together on one clade. The general tree topology was supported by Bootstrap analysis.

## Discussion

Phenotypic differences identified in inter- or intra-species comparisons could be mostly attributed to transcript sequence and expression polymorphisms of their functionally relevant genes. The availability of next-generation sequencing technologies enables us to sequence entire transcriptomes and simultaneously evaluate transcript sequence and expression variation. We applied the RNA-seq technology to examine transcript sequence and expression variations in soybean seeds of nine soybean lines varying in oil composition and content. Our results suggest this is a highly effective approach to the identification of transcript polymorphisms (variation). Compared to whole genome sequencing, RNA-seq represents a genome-reducing technique. The soybean genome is about 1.1 Gb in size with the majority, 59%, consisting mostly of highly repetitive transposable elements [30]. The protein-coding genes account for about 10% of the soybean genome while those expressed in seeds fall to about 6.5%. Thus, RNA-seq represents more than 15-fold enrichment when compared to DNA-sequencing at equal read counts and therefore provides a much more efficient method for identifying sequence variations in expressed soybean seed genes. Even though highly abundant transcripts may reduce efficiency in determining less-abundant transcript sequence, since most of these transcripts are important to seed qualities, knowledge about their expression variation is highly valuable for seed quality improvement. In addition, RNA-seq focuses on the functional components of the genome. While non-transcribed regulatory elements such as promoter and DNA methylation sequences are not captured when using an RNA-sequence approach, their effects can still be measured by transcript accumulation. Transcript SNPs were preferentially located in the active recombination portions of the genome. Higher recombination rates are preferred as recombination breaks down the high linkage disequilibrium (LD) in cultivated soybean varieties, which improves the resolution of genetic maps and facilitates gene mapping. In addition, our sequence data confirm or improve gene models, splice predictions and annotation of the soybean genome.

North American soybean cultivars have a very narrow genetic base. Domestication in Asia, introduction of relatively few landraces to the US and subsequent selection in the breeding process represent three genetic bottlenecks that have reduced the soybean gene pool [6]. Gizlice *et al.*[47] showed that about 35 varieties (28 landraces and 7 first progeny) contribute 95% of alleles found in modern North American soybean cultivars. In this study, we defined the sequence and expression variation of seed transcriptomes from nine soybean lines. Sequencing a relatively small number of lines could capture a large portion of common genotypic variations in a species. In *Arabidopsis*, for example, 20 diverse varieties contained 90% of the common SNPs in this species [48,49]. Similarly, based on entries in dbSNP, 82% of SNPs discovered in our nine lines overlapped with SNPs available from various soybean genotypes. However, sequence variations unique to one line or with lower allele frequencies are often valued in mutant discovery as can be seen with the *FAB2C* allele from FAM94-41.

Seeds are the sites of reserve production and accumulation rendering them the most valuable part of plants and the target for soybean improvement. While a certain percentage of genes expressed in seeds are housekeeping genes and/or are transcribed in multiple tissues, our data appear consistent with previously published expression profiles on seed development [14,50], as many of the here-in-identified genes are strictly related to seed development and are exclusively expressed in seeds. A number of those genes are transcribed at high levels and play major roles for enhancing agronomic traits. For example, we found most of the glycinins and β-conglycinins composing 70% of soybean storage proteins highly expressed in seeds, which is in accord with previously published data [14,31,32,51]. Expression levels of these genes contribute to protein composition and content, and therefore the quality of soy products such as tofu. However, a number of genes whose protein products are not agronomically desirable due to their anti-nutritional effect are expressed in high quantities as well. Among those are genes encoding for protease inhibitors including Bowman-Birk serine protease inhibitors and Kunitz trypsin inhibitors that hamper the digestion of soybean proteins in animals and humans [52-54], and the lipoxygenases involved in oxidation of polyunsaturated fatty acids, which add unpleasant flavors to soybean products [33]. Many of the above mentioned genes show expression variation among the nine lines. For example, the genes coding for glycinin 3 and the most abundant Kunitz trypsin inhibitor are 46-fold and 6-fold less expressed in line R95-1705 compared to the average value from the nine lines. Seven hundred sixty-eight of the 1,075 genes putatively involved in acyl lipid metabolism are expressed in seeds, of which 188 had expression polymorphism in the nine lines including oleosins [35] and caleosins [37]. Line R05-591, for instance, displays a 17-fold decrease in the most expressed caleosin. Transcript expression variations identified in those lines may be of value in the discovery of underlying molecular regulatory mechanisms and the development of effective breeding strategies for seed quality improvement. Additionally, transcript expression variation could potentially be developed into bio-markers for molecular breeding. Although transcription factors are important for plant and seed development, they are more often significantly less expressed than structural proteins and enzymes. The high specificity and sensitivity of RNA-seq will allow us to investigate expression differences of transcription factor genes as well.

SNPs are one of the most widely used DNA sequence markers in eukaryotes. Previously, SNPs were discovered in various soybean cultivars, land races, elite lines and *Glycine soja* using next generation DNA sequencing [18-22] as well as other methods [55-57]. Here we took advantage of RNA-seq and identified 48,792 non-redundant SNPs in 14,840 genes. About 63% (30,535) of SNPs are in CDS regions. Thirty percent (14,372) of them are nonsynonymous, causing amino acid substitutions. The effect of those amino acid changes on protein integrity and function can be predicted with algorithms such as PROVEAN [42], SIFT [58] and PolyPhen-2 [43]. In addition, 125 SNPs introduced premature termination codons and 49 removed stop codons. Seventeen SNPs eliminated the annotated translation initiation codon and 606 SNPs are located at intron splice donor or acceptor sites possibly interfering with transcript splicing. In contrast to SNPs, the effects of 1 and 2 bp indels are more predictable, as the 241 indels found in CDS interrupt the reading frame and protein sequence while the 35 indels identified in splice sites may lead to intron retention and the 4 indels found to eliminate termination codons potentially produce a longer protein. Sequence variations that affect gene function and cause phenotypic variation represent functional (also known as perfect or diagnostic) markers [59,60]. Due to complete linkage with trait phenotypes, functional markers are ideal for marker-assisted breeding. This study identified a large collection of sequence polymorphisms (SNPs and small Indels) that could lead to protein sequence changes and potentially to biological functional changes in genes. These transcript sequence polymorphism could be further developed into semi-functional or functional markers.

The ratio of nonsynonymous to synonymous substitutions (N/S) for all gene models in our nine lines was 0.89, which is substantially lower than the previously published N/S ratio of 1.38 in cultivated soybeans [18]. The N/S ratio for genes expressed in soybean seeds is slightly higher than that in Arabidopsis (0.83) [49], but less than those in sorghum (1.36) [61] and rice (1.2 and 1.29) [62,63]. The differences in these observed ratios might reflect different gene sets, selection criteria, sequence platforms used or allelic expression bias that may be present in RNA-Seq. In rice and soybean, it has been shown that genes with vital functions seem to have significantly lower nonsynonymous-to-synonymous substitution ratios, while genes with non-essential functions such as disease resistance genes have higher ratios [18,63]. Usually, the majority of substitutions are thought to be eliminated by negative (or purifying) selection whereas a small amount of nonsynonymous variants are retained by positive selection under natural conditions. Accordingly, the N/S ratio is normally used to learn about the selection pressure acting on a system. However, soybean lines have been artificially selected by plant breeders, which caused genetic bottlenecks. Therefore, in this situation, N/S ratios may not be used to accurately infer selection pressure [64].

Consistent with the previous reports, we independently identified a number of transcript sequence and expression polymorphisms potentially responsible for altered fatty acid composition. We showed that the *FAB2C* (*SACPD-C*) gene encoding a stearoyl-acyl-carrier-protein desaturase had a putatively damaging SNP in FAM94-41 [44,45]. *FAD3A* encodes a linoleate desaturase that introduces a third double bond in linoleic acid to form linolenic acid. Cardinal *et al.*[65] suggest that lower expression of the *FAD3A* allele in N0304-303-3 is likely to cause a lower level of linolenic acid (18:3) content and is mirrored in our finding. M23 has a 164 kb deleted region containing the fatty acid desaturase gene *FAD2-1A*[66,67] and therefore, as expected, we detected little more than background expression of the *FAD2-1A* gene in M23. Additionally, we detected a coordinated increase in the expression of the four genes comprising the *rhg1* locus in cultivar Jack. The *rhg1* locus has been shown to control the soybean cyst nematode (SCN) resistance trait through copy number variation [8]. Jack is a SCN resistant cultivar and was developed from Fayette, whose rhg1 locus is amplified by 10 fold [46]. We show here that the copy number of the genes in this locus was 10 fold higher than flanking genome sequences, indicating that the coordinated increased expression of those genes in Jack was caused by a copy number amplification.

We generated a valuable collection of sequence and expression variations from these soybean lines, which can be used to address questions in basic and applied soybean research. As mentioned earlier, our sequence polymorphisms are highly applicable for gene mapping approaches and marker-assisted breeding. Moreover, existing QTL data may be compared with our sequence and expression polymorphism data to identify possible candidate genes for traits of interest. For example, 164, 34, 19, 36, 35 and 41 QTLs for oil, palmitic acid, stearic acid, oleic acid, linoleic acid and linolenic acid content are currently (as of Sept 2013) listed in SoyBase (http://soybase.org), respectively, many of which do not have a causal gene assigned to them. Although QTLs usually span large regions containing many genes, genes of interest are limited to those that are transcribed in these regions. The transcript polymorphisms discovered here should greatly facilitate identification of the causal genes and gene variants.

The transcript sequence and expression polymorphisms are also useful for eQTL (expression QTL) analysis to identify DNA variants that change expression levels and patterns of genes [68]. Loci controlling gene expression can act at the transcriptional, co-transcriptional and posttranscriptional level. eQTLs include SNPs resulting in RNA splicing variations, which have been shown to be one of the underlying causes of expression differences and phenotypic diversity. We are in the process of investigating splice variations and identifying line-specific transcript isoforms among the nine soybean genotypes.

## Conclusion

In this study, we sequenced seed transcriptomes from nine soybean genotypes varying in oil composition and content, and provided a comprehensive depiction of transcript accumulation and sequence variation in seeds at gene, pathway and systems levels. We identified a large collection of transcript sequence and/or accumulation polymorphisms that could potentially affect gene functions. Additionally, we developed a variety of data mining strategies and successfully detected a number of genetic variants potentially causing oil composition and SCN resistance changes. Transcriptome sequencing can simultaneously determine two major functional attributes of a gene, transcript sequence and accumulation level. Although transcriptome sequencing is mainly employed to measure transcriptional changes in a single genotype, the proof-of-concept study demonstrates that transcriptome sequencing could offer an effective approach to explore and exploit genetic diversity for discovering sequence and expression markers, gene variants and genome structure variations important to agronomic traits. The collection of transcript polymorphisms identified in this study could serve as highly effective markers for genetic mapping and gene function discovery in soybean seed quality research.

## Methods

### Plant material

All soybean (*Glycine max* (L.) Merrill) lines were grown in the Danforth Center growth chambers with temperature set at 25°C day/ 23°C night, the humidity at 50% and 16-hour daylight at up to 1,000 μmol of supplemental lighting. The plants were watered, fertilized and managed for pests and disease as needed. Seeds at the S6 stage of seed maturation were carefully selected based on seed weight and color, and were harvested for RNA preparation. At the S6 stage, soybean seeds have the highest fresh weight and accumulation levels of transcripts encoding seed storage proteins and fatty acid desaturase. S6 represents one of the most relevant seed developmental stages with respect to storage reserve production [69].

### Oil composition and content

The oil composition of soybean seeds was determined with the Agilent 7890A GC with S/Sl injection and FID detection at the USDA-ARS Plant Genetic Resources Conservation Unit (PGRCU) in Griffin, Georgia. Three single seeds per soybean line were measured with two replications each. The oil content of soybean seeds was determined with the MQC Benchtop NMR Analyser, Oxford Instruments. Six g of seeds per line were measured with two biological and three technical replicates.

### RNA extraction and library construction

Total RNA was isolated as described by Chen and An [70] with minor modification. Nine RNA-seq libraries were constructed and sequenced at Expression Analysis, Inc., Durham NC. RNA-seq libraries were prepared with the TruSeq™ RNA Sample Preparation Kit v2 from Illumina, Inc., San Diego, CA, and 100 bp paired-end reads were generated on the Illumina HiSeq 2000 platform.

### RNA-seq read pre-processing

CASAVA 1.8.2 (Illumina, Inc., San Diego, CA) was used to produce purity filtered reads, which were de-multiplexed and had the adaptors removed using ea-utils-1.1.2 by Expression Analysis, Inc. After evaluating read quality with FastQC (http://www.bioinformatics.babraham.ac.uk/projects/fastqc/), FASTQ/A Trimmer from the FASTX-Toolkit (http://hannonlab.cshl.edu/fastx_toolkit/index.html) was used to remove the last 10 bases of each read leaving 90 bp pair-end reads for the nine genotypes.

### RNA-seq read mapping, transcript assembly and quantification

The free, open-source “Tuxedo” software suite was used in the RNA-seq data analysis [71]. Bowtie version 0.12.8 [72] and TopHat version 1.4.0 [73] were run on each of the samples using the *Glycine max* cv. Williams 82 reference genome [30] and the Gmax_189_annotation from Phytozome version 9.1 (http://www.phytozome.net/) to guide the RNA-seq read alignments. The sequence reads that mapped to the Williams 82 reference genome with no more than two mismatches were used for further analysis. Reads aligned to more than one location on soybean genome were eliminated from the analysis.

Cufflinks version 2.1.1 [74] was run on each sample BAM file to assemble the transcripts. Subsequently Cuffmerge version 2.1.1 was used to produce the merged transcriptome file and Cuffdiff version 2.1.1 to quantify the normalized expression values across nine soybean lines. Genes with a mean expression value across the nine lines of 0.5 FPKM or greater or were expressed in all nine lines were defined as expressed genes to reduce the background noise. Sequences that aligned to the chloroplast and mitochondrial genomes were removed. SAMtools [75] was run on each BAM file to determine read coverage and coverage for each genomic base by each sample. Gene and transcript coordinates from the merged.gtf file from Cuffmerge were used by custom Perl scripts in the calculation of coverage statistics. Z-scores of log_2_-transformed FPKM values for each individual gene in all nine lines were calculated to determine its significance of its expression variation among lines with a Z-score cutoff value of 2.

We applied a computational algorithm that we developed (unpublished) to identify genomic regions that contain adjacent genes coordinately up-regulated or down-regulated in comparison to the other lines. Overall, FPKM values generated in our RNA-Seq expression analysis were used to calculate its Z-Score. Next all expressed genes were ordered by chromosome and position. Samples with three or more genes in a row with Z-Scores of < = - 2 or > =2 are considered down-regulated or up-regulated, respectively. These regions are subject to greater scrutiny in a manual review of the relevant data.

### SNP analysis

Both SAMtools’ mpileup [75] and GATK’s (Genome Analysis Toolkit) Unified Genotyper [76,77] were independently used to call SNPs and small indels across all samples simultaneously. The results were filtered using GATK’s VariantFiltration, where in order to pass, read depth was required to be > =5 reads, SNP quality had to be > =50, and no more than 2 SNPs in a 10 base pair window were allowed. SNPs and indels passing the filtering criteria and that were common to both detection methods were retained.

### Phylogenetic analysis

The nucleotides at 48,792 SNP positions were concatenated and assembled into a single sequence for each soybean line. Subsequently, all assembled nucleotide sequences were aligned based on their chromosome position [78]. SNP positions that are heterozygous in a line, identical in all lines or contain a missing nucleotide in a line were eliminated from the phylogenetic analysis. The assembled nucleotide sequences were used to infer the evolutionary history using the Maximum Likelihood method based on the Tamura-Nei model [79,80]. The tree with the highest log likelihood (-247504.5363) is shown. Initial tree(s) for the heuristic search were obtained automatically by applying Neighbor-Join and BioNJ algorithms to a matrix of pairwise distances estimated using the Maximum Composite Likelihood (MCL) approach, and then selecting the topology with superior log likelihood value. The tree was drawn to scale, with branch lengths measured in the number of substitutions per site. The analysis involved nucleotide sequences for 9 lines. There were a total of 33,164 positions in the final dataset. Evolutionary analyses were conducted in MEGA5 [79].

### Availability of supporting data

RNA-Seq data for nine soybean lines are available under NCBI-GEO series accession no. GSE56297.

## Competing interests

The authors declare that they have no competing interests.

## Authors’ contributions

WG and YQA designed and conducted the experiment, conducted data analysis and prepared the manuscript. YQA conceived of the study and coordinated executing the experiments. EX measured seed oil content. MW measured seed fatty acid composition. RU and PC provided genetic materials. All authors read and approved the final manuscript.

## Supplementary Material

Additional file 1: Figure S1 Accumulative transcript amount of most highly expressed genes in seeds. Genes were sorted by average expression in percent across nine lines. The smallest number of genes (i.e. top expressers) required to reach the accumulative transcript amounts in percent as indicated on the X-axis are shown. **Figure S2.** Distribution of expression variation per line. For each of the 8,037 differentially expressed genes, we determined the line that exhibits the largest expression variation (i.e. absolute Z-score) compared to the average expression across all nine lines. The total count for up-regulated (blue columns) and down-regulated (red columns) genes per line is presented. Click here for file

Additional file 2: Table S1 100 most abundant transcripts in seeds. **Table S2.** Genes showing expression variation across nine lines. **Table S3.** Expression variation by fold-change. **Table S4.** Average expression ratio of genes with and without SNPs that led to pre-mature stop codons. **Table S5.** Possibly damaging non-synonymous SNPs in putative acyl lipid genes. Click here for file
