# Supplementary material for: Identification and characterization of transcript polymorphisms in soybean lines varying in oil composition and content
Source: BMC Genomics. 2014 Apr 23;15:299. doi: 10.1186/1471-2164-15-299 (PMC4023607; doi:10.1186/1471-2164-15-299)

Figure S1: Accumulative transcript amount of most highly expressed genes in seeds

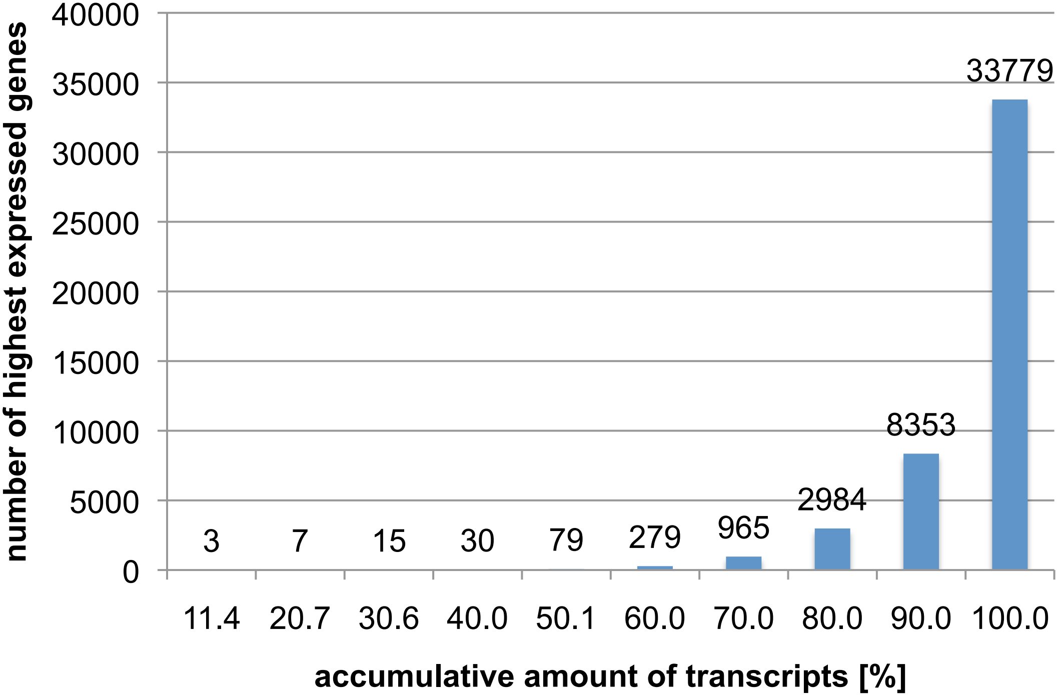

Figure S2: Distribution of expression variation per line

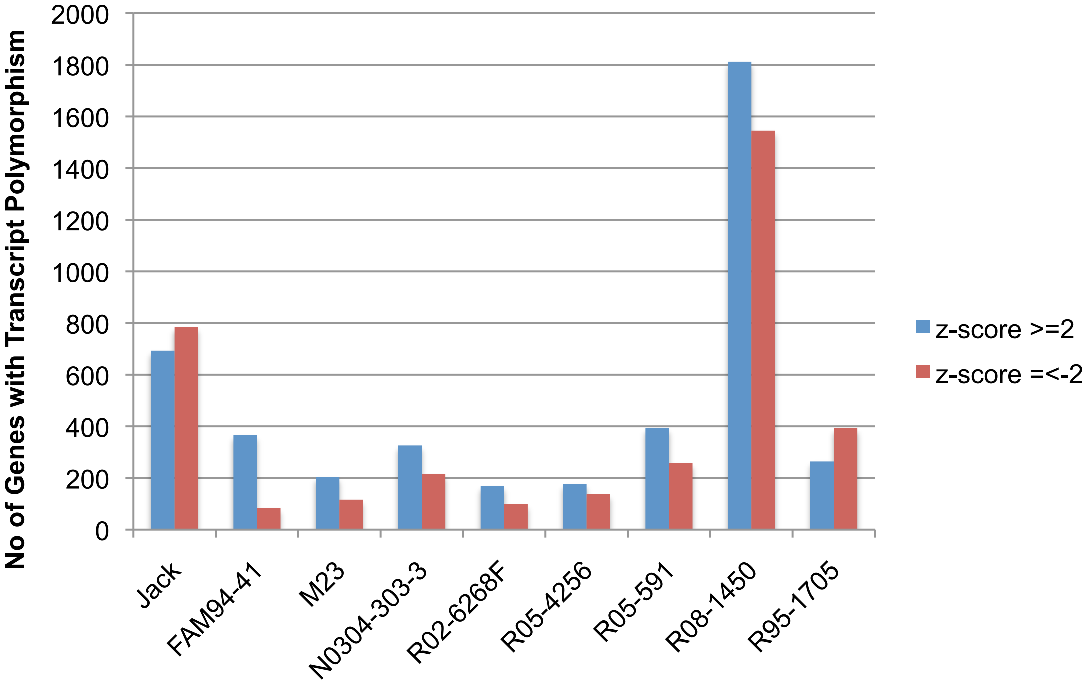

Supplement: Additional file 1: Figure S1 — Accumulative transcript amount of most highly expressed genes in seeds. Genes were sorted by average expression in percent across nine lines. The smallest number of genes (i.e. top expressers) required to reach the accumulative transcript amounts in percent as indicated on the X-axis are shown. Figure S2. Distribution of expression variation per line. For each of the 8,037 differentially expressed genes, we determined the line that exhibits the largest expression variation (i.e. absolute Z-score) compared to the average expression across all nine lines. The total count for up-regulated (blue columns) and down-regulated (red columns) genes per line is presented. [file 1471-2164-15-299-S1.pdf]
